# Added value of quantitative, multiparametric 18F-FDG PET/MRI in the locoregional staging of rectal cancer

**DOI:** 10.1007/s00259-022-05936-0

**Published:** 2022-09-05

**Authors:** Alexander Herold, Christian Wassipaul, Michael Weber, Florian Lindenlaub, Sazan Rasul, Anton Stift, Judith Stift, Marius E. Mayerhoefer, Marcus Hacker, Ahmed Ba-Ssalamah, Alexander R. Haug, Dietmar Tamandl

**Affiliations:** 1 Department of Biomedical Imaging and Image-Guided Therapy, Medical University of Vienna, Währinger Gürtel 18-20, 1090 Vienna, Austria; 2 Department of General Surgery, Medical University of Vienna, Vienna, Austria; 3 Department of Pathology, Medical University of Vienna, Vienna, Austria; 4 INNPATH GmbH, Tirolkliniken, Innsbruck, Austria; 5 Department of Radiology, Memorial Sloan Kettering Cancer Center, New York, NY USA; 6 Christian Doppler Laboratory for Applied Metabolomics, Medical University of Vienna, Vienna, Austria

**Keywords:** PET/MRI, MR, PET, Rectal cancer, Staging

## Abstract

**Purpose:**

The purpose of this study was to determine whether multiparametric positron emission tomography/magnetic resonance imaging (mpPET/MRI) can improve locoregional staging of rectal cancer (RC) and to assess its prognostic value after resection.

**Methods:**

In this retrospective study, 46 patients with primary RC, who underwent multiparametric 18F-fluorodeoxyglucose (FDG) PET/MRI, followed by surgical resection without chemoradiotherapy, were included. Two readers reviewed T- and N- stage, mesorectal involvement, sphincter infiltration, tumor length, and distance from anal verge. In addition, diffusion-weighted imaging (DWI) and PET parameters were extracted from the multiparametric protocol and were compared to radiological staging as well as to the histopathological reference standard. Clinical and imaging follow-up was systematically assessed for tumor recurrence and death.

**Results:**

Locally advanced rectal cancers (LARC) exhibited significantly higher metabolic tumor volume (MTV, AUC 0.74 [95% CI 0.59–0.89], *p* = 0.004) and total lesion glycolysis (TLG, AUC 0.70 [95% CI 0.53–0.87], *p* = 0.022) compared to early tumors. T-stage was associated with MTV (AUC 0.70 [95% CI 0.54–0.85], *p* = 0.021), while N-stage was better assessed using anatomical MRI sequences (AUC 0.72 [95% CI 0.539–0.894], *p* = 0.032). In the multivariate regression analysis, depending on the model, both anatomical MRI sequences and MTV/TLG were capable of detecting LARC. Combining anatomical MRI stage and MTV/TLG led to a superior diagnostic performance for detecting LARC (AUC 0.81, [95% CI 0.68–0.94], *p* < 0.001). In the survival analysis, MTV was independently associated with overall survival (HR 1.05 [95% CI 1.01–1.10], *p* = 0.044).

**Conclusion:**

Multiparametric PET-MRI can improve identification of locally advanced tumors and, hence, help in treatment stratification. It provides additional information on RC tumor biology and may have prognostic value.

**Supplementary Information:**

The online version contains supplementary material available at 10.1007/s00259-022-05936-0.

## Introduction

With over 1.9 million new cases and 935,000 deaths in 2020, colorectal cancer is the third most common cancer and second leading cause of cancer-related death worldwide, with rectal cancer (RC) accounting for about one-third of patients and associated deaths [1]. Overall declines in colorectal cancer incidence mask the increasing rates of early-onset colorectal cancer among patients under 50 years of age, largely driven by RC [2].

Accurate primary locoregional staging in RC is pivotal for optimal multidisciplinary disease management, as well as prognostication. Initial disease stage is the most important predictive factor for overall survival (OS) since 5-year survival rate decreases from 91% in stage I to 72% in locally advanced rectal cancers (LARC; T ≥ 3, N +), and drastically to 15% in stage IV RC [3, 4]. In the last few decades, advances in surgical techniques, including standardized total mesorectal excision (TME), chemotherapy, radiotherapy (RT) and chemoradiotherapy (CRT), as well as a better understanding of surgical oncology, have led to a significant reduction in locoregional recurrence rates and longer OS [5]. In LARC, the standard of care consists of neoadjuvant CRT followed by surgery, which has improved control of local disease with local recurrence rates of 5–7% [6, 7]. However, recurrence of distant disease remains high. Recently, the results of the RAPIDO and PRODIGE-23 phase III clinical trials using total neoadjuvant chemo(radio-)therapy in LARC have demonstrated promising results, improving disease free survival and overall survival [8, 9]. Of note, the majority of oncologic therapy in those regimens (i.e., CRT) is administered before any surgical histology becomes available. Thus, the treatment decision heavily relies on accurate staging information and not post-resection histopathology. In addition, precise staging has an economic impact since treatment costs increase exponentially from early to late stages [10, 11].

According to the European Society of Gastrointestinal and Abdominal Radiology, as well as the National Comprehensive Cancer Network, pelvic MRI remains the gold standard for locoregional staging of RC [12, 13]. MRI delivers anatomical tumor information with depiction of T-staging, involvement of the mesorectal fascia (MRF), extramural vascular invasion (EMVI), and locoregional lymph node metastases [14]. However, despite being superior to other imaging modalities, the ability of MRI to predict the exact tumor stage remains suboptimal, primarily due to poor detection of metastatic lymph nodes and differentiation between T2 and T3 tumors [15].

Positron emission tomography/magnetic resonance imaging (PET/MRI) enables the simultaneous acquisition of metabolic information from PET along with a superb MRI anatomic layout and functional data from dynamic contrast-enhanced MRI and DWI, reflecting the pathophysiologic characteristics of the tumor [16]. Furthermore, PET/MRI has shown its ability to predict even more complex tumor features such as tumor phenotype and circulating miRNA [17, 18]. Where available, it has emerged as a “one-stop shop” examination, not only for colorectal cancer, but also for various other oncologic diseases [19–23]. However, sensitivity of small lung nodule detection remains lower than in standard of care imaging [24]. Particularly for local staging of RC, PET/MRI, due to the higher soft-tissue signal and contrast-to-noise ratios of MRI, has been shown to outperform both positron emission tomography/computed tomography (PET/CT) and MRI alone [3, 25, 26]. Nevertheless, the exact impact on the accuracy of locoregional staging remains unclear, since, in most studies, many patients with RC have received neoadjuvant CRT, thus altering the T, N, and MRF status in the resected specimen compared to treatment-naïve tissue.

Therefore, the aim of this study was to determine whether locoregional staging and tumor assessment can be improved by multiparametric PET/MRI compared to MRI alone, in patients with early and intermediate-stage RC, who underwent resection without neoadjuvant CRT. Moreover, the prognostic value of mpPET/MRI parameters on long-term survival was evaluated.

## Materials and methods

Patients with histopathologically confirmed, untreated adenocarcinoma of the rectum who underwent routine clinical 18F-fluorodeoxyglucose (FDG) PET/MRI for staging of primary RC between July 2015 and December 2020 were potentially eligible for this study. This study was approved by the local institutional review board of the Medical University of Vienna (IRB-No. 1403/2015) and performed in accordance with the Declaration of Helsinki. Written, informed consent was waived for this retrospective analysis. Low- to intermediate-risk patients, according to current European Society of Medical Oncology guidelines [27] (T < 3 for low tumors, up to T3ab for middle or high tumors, N0, but N1 allowed if upper-third tumors, MRF not involved, no EMVI), who had been treated with surgical resection without neoadjuvant chemotherapy or chemoradiotherapy, were selected via a hospital information system database search. Treatment decisions were based on routine multidisciplinary tumor boards after reviewing all available imaging material at the time of diagnosis. Exclusion criteria were (i) age < 18 years, (ii) pretreatment with chemotherapy or chemoradiotherapy, (iii) surgical resection not performed, (iv) poor examination quality, and (v) blood glucose level > 150 mg/dl. From an initial cohort of 119 patients who had undergone PET/MRI for primary RC, 57 patients were excluded due to neoadjuvant treatment, six patients did not undergo surgery, five studies had poor image quality or incomplete studies, and, in five patients, histopathological data was incomplete. The final study group consisted of 46 patients who fulfilled all the criteria.

### PET/MRI protocol

All patients underwent multiparametric PET/MRI examinations using a fully integrated 3.0 Tesla PET/MRI system (Biograph mMR; Siemens, Erlangen, Germany). Patients fasted at least 4 h before imaging; the glucose cutoff level tolerable for the scan was 150 mg/dl. PET image acquisition was performed after the administration of body weight-adapted 18F-FDG (median 237 ± 38 MBq) during the dedicated pelvic MRI acquisition and at 3–5 min/bed position for the whole-body (WB) study. Image reconstruction was acquired using the point spread function-based algorithm “High-Definition PET Reconstruction” with three iterations and 21 subsets. Rectal MRI sequences were performed immediately after 18F-FDG injection and included at least axial, axial oblique, coronal and sagittal high resolution T2w Turbo spin echo (TSE) sequences and axial diffusion-weighted (DWI) sequences with apparent diffusion coefficient (ADC) maps. Approximately 30–40 min after injection, after completion of the rectal MRI, a co-acquired WB MRI scan was performed, using a standard protocol including a WB axial, two-point Dixon, three-dimensional, volume-interpolated, T1-weighted breath-hold MRI sequence (VIBE) for attenuation correction and coronal, T2-weighted, HASTE (half-Fourier acquisition single-shot) turbo spin-echo sequences. These sequences were acquired from skull and moved to mid thighs and were not subject to analysis for the current study. The PET/MRI protocol has been summarized in Table S1. A standard dose of 20 mg N-Butylscopolamin s.c. and 50 ml of rectal ultrasound gel for improved lesion visualization was applied as per institutional standards.

### Image analysis and interpretation

Two readers, one board-certified nuclear medicine physician and a board-certified radiologist, performed image analysis during the routine staging workflow, using a dedicated workstation that included post-processing software (Syngo.via, Siemens Healthineers, Erlangen, Germany).

For study purposes, retrospective assessment of MRI-based T- and N-staging (mrT- and mrN-stage), mesorectal fascia involvement, sphincter infiltration, tumor length, distance from the anal verge, and EMVI was performed by two gastrointestinal radiologists with nine and 25 years of experience. Only the pelvic MRI portion was evaluated for locoregional staging using T2-weighted and DWI-sequences in accordance with current guidelines for rectal MRI [13]. The readers were blinded to the PET-data and histopathology results. In case of discrepancy of T- or N-staging, a decision was made by consensus. Pathologic lymph nodes were defined as > 5 mm, with round or indistinct margins, as well as heterogenous signal intensity on T2w-sequences, as detailed in the guidelines [13]. Apart from T- and N-staging, it was assessed whether tumors were locally advanced (T ≥ 3 *and/or* N +), since this adverse prognostic feature is used by many clinicians and guidelines to trigger neoadjuvant treatment (although, in oncologic guidelines, this is not always an automatic prerequisite, depending on tumor level [27]; see inclusion criteria).

In a separate process, one radiologist with 2 years of experience quantitatively analyzed DWI parameters, which had been extracted and processed in a dedicated viewer software (OsiriX © Pixmeo Sarl 2020). Regions of interests were manually drawn at three adjacent representative levels of the tumor and the mean value was used for further analysis.

Quantitative PET parameters were calculated from the pelvic PET portion of the WB scan (60 min post injection), extracting the maximum, peak, and mean standardized uptake value (SUV_max_, SUV_peak,_ and SUV_mean_), as well as metabolic tumor volume (MTV) and total lesion glycolysis (TLG), at a fixed SUV threshold of 4.0. They were compared to the same parameters obtained during the last 10 min of the pelvic PET acquisition (30 min post injection). All cases were reviewed in a consensus meeting by a nuclear medicine physician with 16 years of experience in hybrid oncological imaging.

### Standard of reference

Histopathologic findings of the surgical specimen were used as the reference standard.

The following unfavorable prognostic features were defined based on histology: (i) T-Stage ≥ pT3a, (ii) N-Stage ≥ pN1, (iii) locally advanced tumor stage (pT ≥ 3a, *and/or* pN +), (iv) lymph node ratio (LNR) ≥ 0.1, (v) positive microscopic lymphovascular invasion (pL1), and (vi) tumor grading > 2 (pG > 2). Microscopic vascular invasion, perineural invasion, MRF status, and EMVI were not considered due to the low prevalence (*n* ≤ 2) of these features in this cohort of early- to intermediate-stage tumors.

Clinical follow-up data, including medical reports, laboratory results including tumor markers, physical examinations, and follow-up imaging, were screened for the assessment of survival.

### Statistical analysis

All calculations were performed using SPSS (SPSS Inc, Version 27). Descriptive statistics were performed for patient and tumor characteristics, as well as for parameters obtained by PET/MRI using median and ranges. Parametric data extracted from DWI sequences (ADC) were analyzed and median, mean, 90th percentile, and interquartile range values were calculated.

Receiver operating curve (ROC) analysis of mpPET/MRI parameters was conducted with regard to unfavorable histologic features as described above. Area under the curve (AUC) with 95% confidence intervals (95% CI) and *p*-values were assessed for each PET and MRI parameter. Optimal sensitivity and specificity levels were determined using the Youden index. Uni- and multivariate binary logistic regression analysis was performed for correlation with clinical and histological features. A stepwise forward inclusion model using likelihood ratios was chosen, and features with a *p*-value of ≤ 0.1 on univariate analysis were considered for two multivariate models. Model 1 included all multiparametric PET/MRI parameters and anatomical MRI sequences; model 2 included only quantitative DWI and PET parameters (SUV_max_, SUV_mean_, SUV_peak_, MTV, TLG, and ADC). A heatmap of the calculated AUC values was generated using the ComplexHeatmap R package in R version 4.1.2. Rows and columns were arranged using complete-linkage clustering based on Euclidean distances.

Sensitivity, specificity, positive predictive value (PPV), and negative predictive value (NPV) of T- and N-stages derived from the anatomical MRI sequences, as well as from the mpPET/MRI model, were calculated in relation to the gold standard, using either cross tables of MRI parameters or ROC curves for continuous variables of DWI and PET. Diagnostic performance was compared using the McNemar test. Survival analysis was performed using a Cox-proportional hazard model with multivariate corrections, as well as the log-rank test, from the date of PET/MRI to the last known visit or death of the patient. A value of *p* < 0.05 indicated statistical significance.

## Results

### Demographics

Forty-six patients, including 14 women and 32 men (median age 66 years, range 38–84) with adenocarcinoma of the rectum, were identified who fulfilled the inclusion criteria. All patients subsequently had radical surgical resection with clear margins and histopathological workup. No patient had received neoadjuvant CRT or systemic chemotherapy. Details on patient demographics and tumor stage are depicted in Table 1.

**Table 1:** Demographic and tumor-related characteristics

Characteristics	All (*n* = 46)	LARC (*n* = 24)	noLARC (*n* = 22)	*P value*
Age (years)	66 (38–84)	69 (39–84)	63 (38–82)	*P* = 0.327
Gender (*n*, %)				
Female	12 (26)	6 (25)	6 (27.3)	*P* = 0.861
Male	34 (74)	18 (75)	16 (72.7)	
Body mass index (kg/m^2^)	24 (14–33.9)	23.9 (18–34)	24.5 (14–30.8)	*P* = 0.457
Grading				
1	1 (2.2)	0 (0)	1 (4.8)	*P* = 0.153
2	36 (78.3)	17 (77.3)	19 (90.4)	
3	6 (13)	5 (22.7)	1 (4.8)	
n.a	3 (6.5)			
T-stage (*n*, %)				
1	7 (15.2)	1 (4.2)	6 (27.3)	*P* < *0.001*
2	20 (43.5)	4 (16.7)	16 (72.7)	
3	19 (41.3)	19 (79.1)	0 (0)	
N-stage (*n*, %)				
0	34 (73.9)	13 (54.3)	21 (100)	*P* = *0.032*
1	7 (15.2)	7 (29.1)	0 (0)	
2	4 (8.7)	4 (16.6)	0 (0)	
n.a	1 (2.2)			
LNR (median, range)	0 (0–0.71)	0 (0–0.71)	0 (0–0)	*P* = *0.008*
< 0.1 (*n*, %)	38 (82.6)	17 (73.9)	21 (100)	*P* = *0.012*
≥ 0.1 (*n*, %)	6 (13.6)	6 (26.1)	0 (0)	
n.a. (*n*, %)	2 (4.3)			
Microscopic lymphatic invasion (*n*, %)				
0	30 (65.2)	14 (60.9)	16 (84.2)	*P* = 0.096
1	12 (26.1)	9 (39.1)	3 (15.8)	
n.a	4 (8.7)			
Localization (*n*, %)				
Lower rectum	8 (17.4)	3 (12)	5 (23.8)	*P* = 0.518
Mid rectum	23 (50)	14 (56)	9 (42.9)	
Upper rectum	15 (32.6)	8 (32)	7 (33.3)	
Tumor size (cm)	3.7 (1.6–10)	3.9 (1.9–6.6)	3.1 (1.6–10)	*P* = 0.436
Distance from anal verge (cm)	9.1 (2.5–11.5)	8.9 (2.7–11.4)	9.4 (2.5–11.5)	*P* = 0.504

All data are presented as median values and ranges, or absolute frequency and percentage. *LARC*, locally advanced rectal cancer (pT ≥ 3 *and/or* pN +); *LNR*, lymph node ratio; *n.a*., not available

### Analysis of mpPET/MRI parameters with regard to unfavorable tumor features

**Fig. 1:**
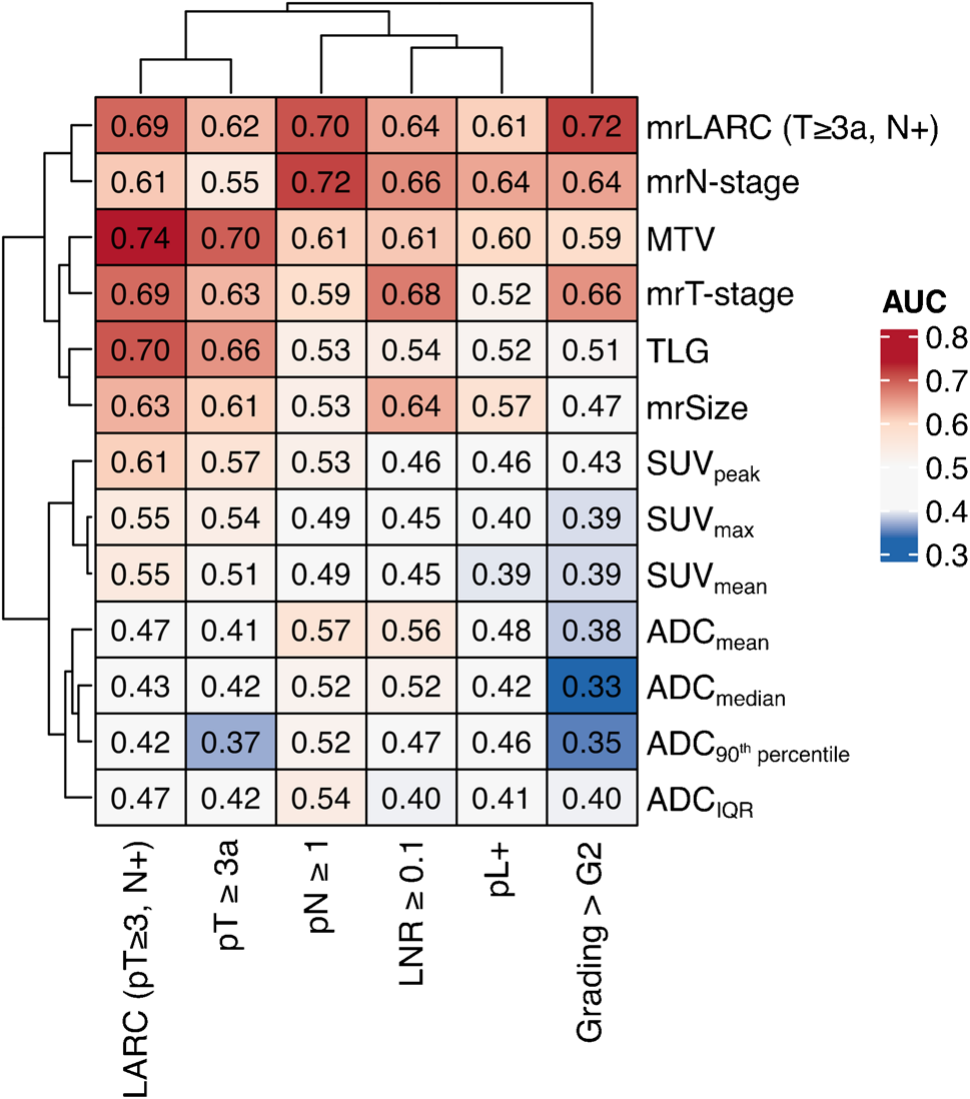
Results of the ROC curve analysis of individual mpPET/MRI parameters (*y*-axis) compared to unfavorable histologic features (*x*-axis). Parameters are grouped with regard to similarity of features and tendency of AUC curves. Row and column dendrograms represent complete-linkage clustering based on Euclidean distances. LARC, locally advanced rectal cancer (pT ≥ 3 *and/or* N ≥ 1); LNR, lymph node ratio; MTV, metabolic tumor volume; TLG, total lesion glycolysis; ADC, apparent diffusion coefficient; SUV, standardized uptake value

Anatomical and quantitative DWI- and PET-derived parameters were compared for unfavorable tumor features using ROC curve analysis; the results are summarized in Fig. 1. Among all PET/MRI parameters, MTV and TLG showed the highest diagnostic performance in identifying LARC (AUC values of the ROC curve of 0.74 [95% CI 0.59–0.89], *p* = 0.004 for MTV and 0.70 [95% CI 0.53–0.87], *p* = 0.022 for TLG, respectively) as well as increased T-stage ≥ pT3a (AUC 0.70 [95% CI 0.54–0.85], *p* = 0.021 for MTV). DWI-derived parameters were not significantly associated with unfavorable features. Nodal stage (pN) was best assessed using anatomical MRI sequences (mrN-stage, AUC 0.72 [95% CI 0.54–0.89], *p* = 0.032), while none of the PET and DWI features were associated with pN.

Subsequent multivariate binary logistic regression analysis of mpPET/MRI parameters demonstrated an independent association of pT- and pLARC-status to the LARC status assessed with MRI, and a strong association of mrN- and pN-status (HR 6.22, 95% CI 1.33–29.0, *p* = 0.020). The quantitative PET and ADC parameters did not show any association to those histopathological characteristics, if included in the model together with the anatomical MRI assessment (model 1, Table 2). In a separate model (model 2), only the quantitative DWI and PET parameters were compared, and MTV demonstrated an independent prediction of pLARC status (HR 1.03, 95% CI 1.00–1.06, *p* = 0.041). For pT- and pN-status, no significant association was found in model 2.

**Table 2:** Multivariate binary logistic regression analysis of mpPET/MRI parameters and histopathologic characteristics

Model 1						
Histopathological characteristics	mpPET/MRI parameters	B	HR	95% CI		*P value*
				lower	upper	
T-Stage (≥ pT3)						
	mrLARC	1.409	4.091	1.036	16.152	*P* = *0.044*
LARC (pT ≥ 3, N +)						
	mrLARC	2.097	8.143	1.958	33.867	*P* = *0.004*
N-Stage (pN ≥ 1)						
	mrN-Stage	1.828	6.222	1.334	29.013	*P* = *0.020*
Model 2						
Histopathological characteristics	mpPET/MRI parameters	B	HR	95% CI		*P value*
				lower	upper	
LARC (pT ≥ 3, N +)						
	MTV	0.031	1.032	1.001	1.063	*P* = *0.041*

Model 1 includes all multiparametric PET/MRI parameters and anatomical MRI sequences, model 2 includes SUV_max_, SUV_mean_, SUV_peak_, MTV, TLG, and ADC. B, Regression coefficient; HR, hazard ratio; 95% CI, 95% confidence interval; MTV, metabolic tumor volume; LARC, locally advanced rectal cancer

### Early vs. standard PET acquisition in relation to histopathological features

Mean time of measurement was 59 ± 5 min after FDG application for the standard assessment of pelvic FDG-uptake, vs. 30.9 ± 3 min for the early assessment, which was measured toward the end of the dedicated pelvic PET/MRI part. All PET parameters yielded a significant increase between the early and standard time point (e.g., MTV 9.7 vs. 18.2 mL, *p* < 0.001 or SUV_max_ 9.2 vs. 14.9, *p* < 0.001). None of the early quantitative PET parameters was associated with the defined unfavorable pathological features, compared to when the standard PET acquisition was used, with also inferior AUC values (e.g., AUC MTV_early_ vs. pLARC 0.636, 95% CI 0.45–0.822, *p* = 0.154). The slope of increase between the early and late PET signal also lacked any association with tumor biology.

### Diagnostic performance of anatomical MRI sequences compared to quantitative PET/MRI parameters

For the comparison of anatomical MRI staging and staging with mpPET/MRI, only the unfavorable histopathologic characteristics T-stage, N-stage, and LARC were chosen since those could be assessed in the MRI portion, as opposed to grading, lymphovascular invasion, and LNR.

MRI correctly identified T-stage in 27/46 patients (58.7%), T1 in 5/7 (71.4%), T2 in 11/20 (55%), and T3 in 11/19 patients (57.9%), respectively. MRI overstaged T-stage in 10 patients (pT1 was staged as mrT2 and mrT3ab in one case each, and eight pT2 tumors were staged mrT3ab) and understaged in nine patients (one pT2 was staged as mrT1 and eight pT3 tumors were staged as mrT2). Mesorectal lymphadenopathy was diagnosed upon histopathology in 11/45 patients (24.4%). In one patient, no histopathological information on nodal status was available due to a local excision of a low T1 tumor and a lack of a TME. MRI identified N-status (negative vs. positive) correctly in 32/45 patients (71.1%) and falsely interpreted 10 patients as node-positive and three patients as false-negative, respectively. LARC (pT ≥ 3 *and/or* pN +) was diagnosed by pathology in 24/46 patients (52.2%). MRI identified 32/46 (69.6%) correctly as either LARC or non-LARC, overstaged nine and understaged five tumors, respectively. Sensitivity, specificity, PPV, and NPV values are depicted in Table 3. The diagnostic performance of other PET/MRI parameters has been summarized in Table S2.

**Table 3:** Diagnostic performance of MRI vs. multiparametric PET/MRI in the primary staging of RC compared to histopathology

MRI vs. histopathology								
Histopathological characteristics	MRI parameters	Sens.	Spec.	PPV	NPV	AUC	95% CI	*P Value*
T-Stage (pT≥3a)	mrT-Stage	0.55	0.654	0.55	0.654	0.629	0.467-0.791	P = 0.138
N-Stage (pN≥1)	mrN-Stage	0.727	0.706	0.444	0.889	0.717	0.539-0.894	*P = 0.032*
LARC (pT≥3a, pN+)	mrT≥3a *and/or* mrN+	0.791	0.591	0.678	0.722	0.691	0.538-0.819	*P = 0.026*
PET/ADC parameters vs. histopathology								
Histopathological characteristics	PET/MRI parameters	Sens.	Spec.	PPV	NPV	AUC	95% CI	*P Value*
T-Stage (pT≥3a)	MTV	0.9	0.577	0.621	0.882	0.695	0.540-0.851	*P = 0.021*
	TLG	0.947	0.435	0.60	0.917	0.657	0.489-0.824	P = 0.083
	SUV_max_	0.895	0.348	0.531	0.800	0.540	0.361-0.719	P = 0.658
N-Stage (pN≥1)	MTV	0.818	0.471	0.344	0.937	0.609	0.441-0.776	P = 0.254
	TLG	0.727	0.400	0.333	0.909	0.533	0.351-0.716	P = 0.731
	SUV_max_	0.545	0.467	0.261	0.722	0.490	0.299-0.680	P = 0.916
LARC (pT≥3a, pN+)	MTV	0.917	0.682	0.759	0.882	0.740	0.588-0.893	*P = 0.004*
	TLG	0.957	0.579	0.733	0.917	0.703	0.534-0.871	*P = 0.022*
	SUV_max_	0.565	0.474	0.541	0.444	0.547	0.370-0.725	P = 0.594
Combined PET and MRI parameters vs. histopathology								
Histopathological characteristics	PET/MRI parameters	Sens.	Spec.	PPV	NPV	AUC	95% CI	*P Value*
T-Stage (pT≥3a)	mrT-Stage*MTV	0.9	0.5	0.581	0.867	0.716	0.567 -0.866	*P = 0.013*
	mrT-Stage*TLG	0.947	0.435	0.581	0.909	0.711	0.554 -0.867	*P = 0.020*
N-Stage (pN≥1)	mrN-Stage*MTV	0.636	0.765	0.467	0.867	0.77	0.628 -0.912	*P = 0.008*
	mrN-Stage*TLG	0.636	0.733	0.467	0.846	0.739	0.582 -0.897	*P = 0.020*
LARC (pT≥3a, pN+)	mrLARC*MTV	0.958	0.5	0.676	0.917	0.808	0.677 -0.939	*P < 0.001*
	mrLARC*TLG	0.956	0.474	0.688	0.9	0.811	0.672 -0.951	*P = 0.001*

*combined with; *Sens*, sensitivity; *Spec*, specificity; *PPV*, positive predictive value; *NPV*, negative predictive value; *AUC*, area under the curve; *CI*, confidence interval; *MTV*, metabolic tumor volume; *TLG*, total lesion glycolysis;  *SUV*_*max*_, maximum standard uptake value; *LARC*, locally advanced rectal cancer

Regarding T-stage, MTV demonstrated a higher sensitivity and a similar specificity compared to MRI T-stage, although this was not statistically significant (90.0% vs. 55.0%, and 57.7% vs. 65.4%, AUC 0.695 vs. 0.629, *p* = 0.396); similar observations were made for TLG. For N-stage, MTV and TLG had a similar sensitivity compared to MRI N-stage; however, specificity was decreased vs. MRI (47.1% for MTV and 40.0% for TLG, compared to 70.6% for MRI, *p* = 0.022). MTV had a higher sensitivity and specificity than MRI to detect LARC, although this was not statistically significant (sensitivity of 91.7% vs. 79.1%, *p* = 0.375 and specificity of 68.2% vs. 59.1%, AUC 0.740 vs. 0.691, *p* = 0.645); results were similar for TLG. Optimal thresholds for differentiating LARC from Non-LARC were 8 for MTV and 48 for TLG, respectively, according to the Youden index.

When combining anatomical MRI assessment and quantitative PET parameters, the sensitivity to detect LARC could be improved to 95.8%, at the cost of a lower specificity of 50.0% (AUC 0.808 [95% CI 0.677–0.939], *p* < 0.001), with a significant difference from MRI assessment alone (AUC 0.691[95% CI 0.538–0.819], *p*-value of ROC comparison = 0.008).

### Survival analysis

**Fig. 2:**
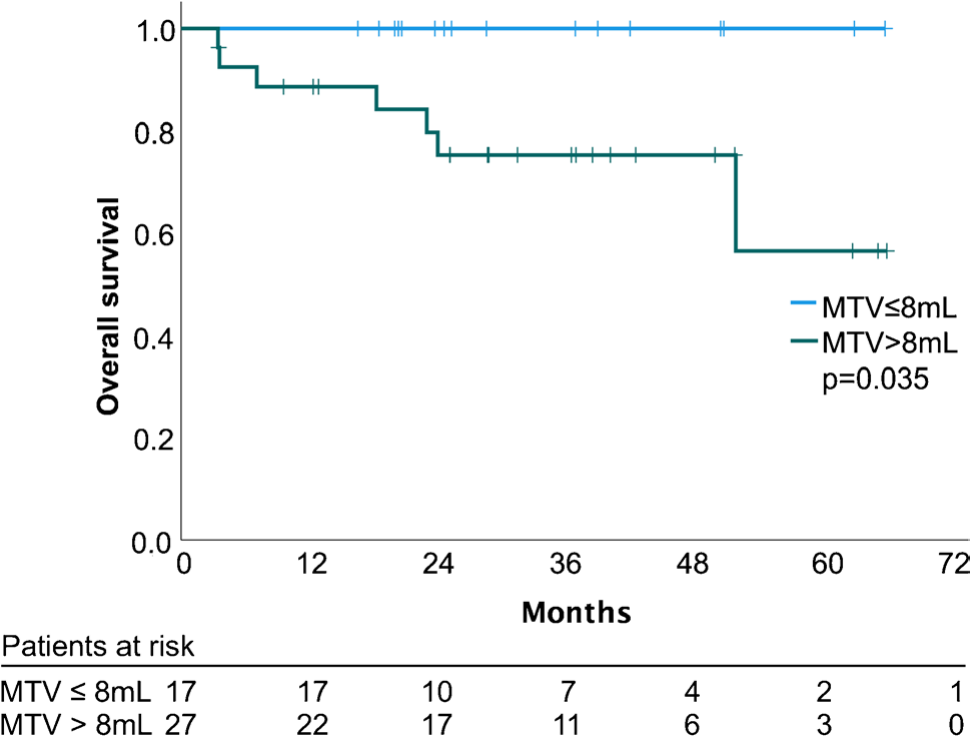
Kaplan–Meier plot of overall survival of rectal cancer patients stratified by metabolic tumor volume

Median follow up was 36.4 months (95% CI 26.5–46.3 months), with two patients lost to follow-up, and thus, not included in the survival analysis. During the observation period, seven patients died, yielding a mean OS of 54.8 months (95% CI 48.0–61.5 months). There were no immediate deaths within 90 days of resection. As expected from tumor biology, patients with pT ≥ 3a had an inferior survival to patients with pT1/2 (mean OS 48.7, 95% CI 38.0–59.4 months vs. 63.5, 95% CI 59.1–67.8 months, *p* = 0.049); however, nodal status did not show an association with poor OS (mean 45.8, 95% CI 30.5–61.1 months vs. 57.0, 95% CI 49.3–64.7 months, *p* = 0.169). Patients with LARC had a worse OS compared to patients who did not have LARC, with a mean survival of 48.1 months (95% CI 38.1–58.3), vs. 65.7 months (*p* = 0.015). In the multivariate Cox regression analysis, MTV remained the only parameter independently associated with OS (HR 1.05 [95% CI 1.01–1.10], *p* = 0.044). Mean survival was 51.7 months (± 21.2 months) for patients with an MTV of greater than 8 mL, while no deaths occurred in patients with an MTV ≤ 8 mL (*p* = 0.035, Fig. 2).

## Discussion

The goal of this retrospective study was to analyze the value of multiparametric PET/MRI in the staging of primary RC without preoperative treatment to gain histologically correlated metabolic and functional information that could potentially improve locoregional staging, and thus, influence treatment stratification. The results of our study demonstrate an association between quantitative PET parameters and unfavorable tumor features, leading to an improved identification of locally advanced tumors by the combination of PET and MRI, possibly influencing treatment stratification and providing predictive information.

The treatment of non-metastatic rectal cancer is heavily driven by the local tumor stage and adverse locoregional tumor features, which tend to increase the likelihood of local and distant recurrence. However, major limitations of the current imaging gold standard, MRI, include poor performance in differentiation between T2 and T3ab stage, mainly due to the desmoplastic reaction of the adjacent mesorectal fat, with reported sensitivities and specificities of 43–72% and 76–94%, respectively [25, 28], which concurs with our results. Although very similar 5-year survival rates in pT3a and pT2 have been reported [29], understaging may lead to omission of RT or CRT and an increase of local relapse, while overstaging may lead to unnecessary treatment and functional consequences. Another major limitation of MRI is its performance in the detection of positive lymph nodes with reported sensitivities of 56–85% and specificities of 54–94%, respectively [30, 31]. Although the combination with morphologic criteria has improved the diagnostic performance [31], it is still subpar given how important treatment decisions, such as total neoadjuvant therapy, rely on its accuracy.

Functional MRI imaging techniques, namely DWI, may contribute valuable, additional information in rectal cancer imaging, particularly regarding therapy response assessment. While DWI is recommended by radiological guidelines [13] due to its proven benefit for tumor detection, as well as evaluation of treatment response, it appears to play only a minor role in the primary staging of rectal cancer. However, quantification of diffusion properties and expressing these properties as an apparent diffusion coefficient could potentially be used as imaging biomarkers of tumor aggressiveness. Although not statistically significant, lower ADC values were associated with poorly differentiated tumors in our study. A recent study shows a similar trend toward low ADC values in high-grade tumors [32].

**Fig. 3:**
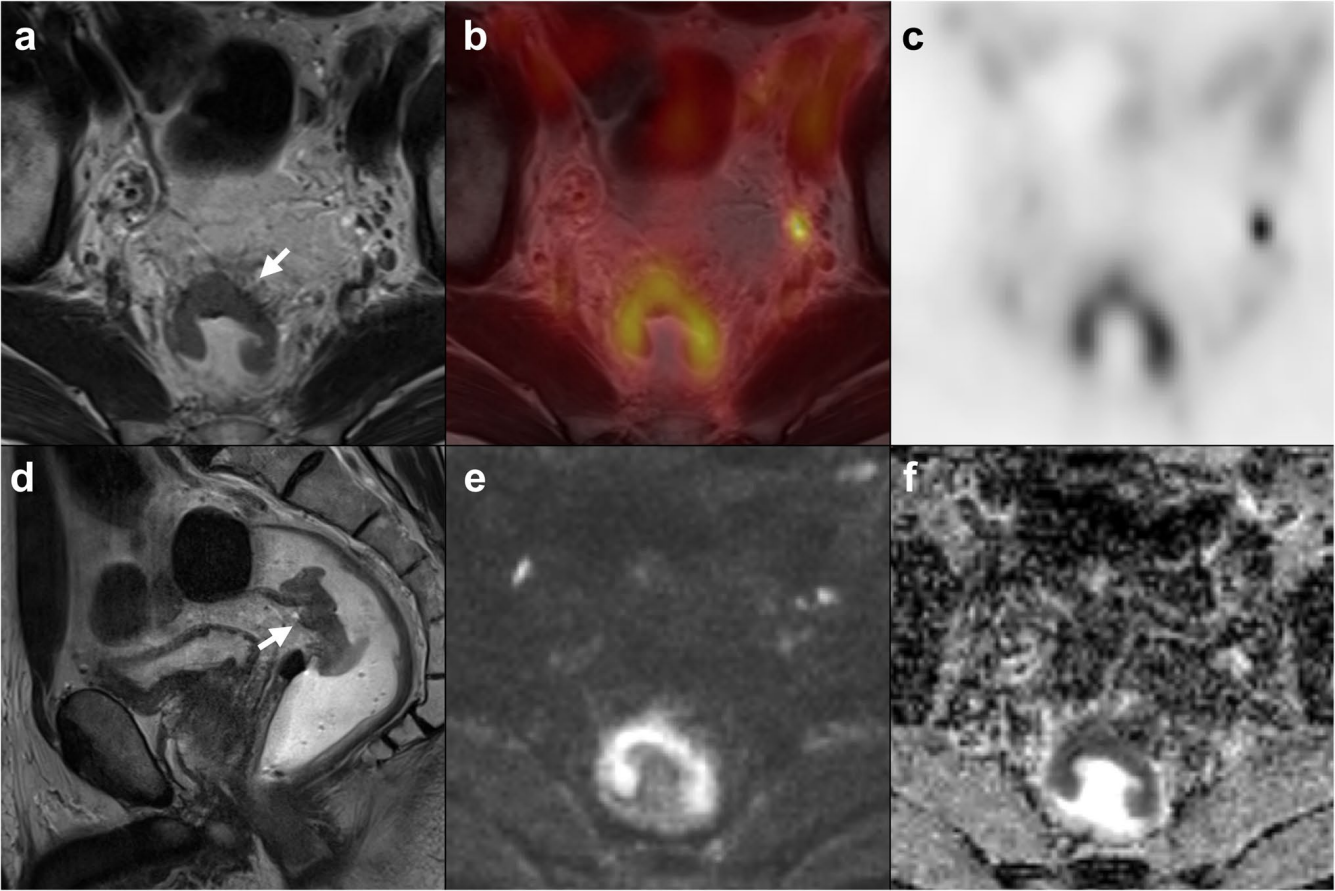
Example of a borderline T2/T3ab tumor based on MRI imaging. Axial (**a**) and sagittal (**d**) high-resolution T2w-MRI images, axial fused PET/MRI (**b**), axial PET (**c**), axial diffusion-weighted imaging (b800) (**e**), and ADC map (**f**). On the axial MRI images, desmoplastic reaction of mesorectal fat adjacent to the tumor (arrow) is appreciated, representing early T3 or T2 stage. PET yielded a metabolic tumor volume of 27.7 mL, which is above the threshold of 8 mL, and thus, favored a T3 stage, which was confirmed upon histopathology (pT3a)

**Fig. 4:**
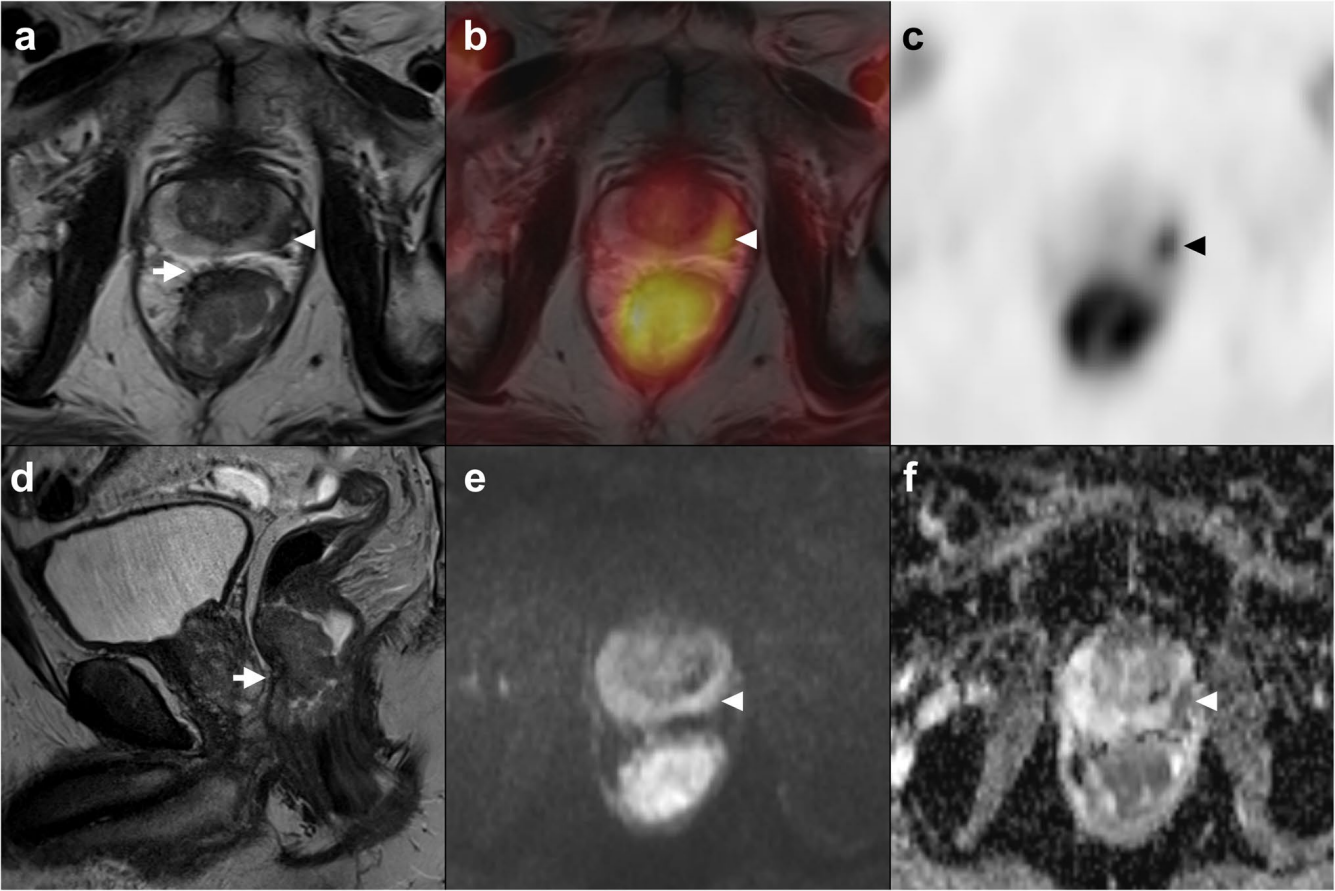
Axial (**a**) and sagittal (**d**) high-resolution T2w-MRI images, axial fused PET/MR (**b**), axial PET (**c**), axial diffusion-weighted imaging (b800) (**e**), and ADC map (**f**). Patient with simultaneous rectal and coincidentally detected FDG-avid prostate cancer (arrowhead, Gleason score 5 + 3, multicentric, pT3a, N0). T2w-images indicate invasion into the mesorectum (mrT3ab). PET yielded a metabolic tumor volume of 21.8 mL, also favoring a T3 stage. Histopathology confirmed pT3

**Fig. 5:**
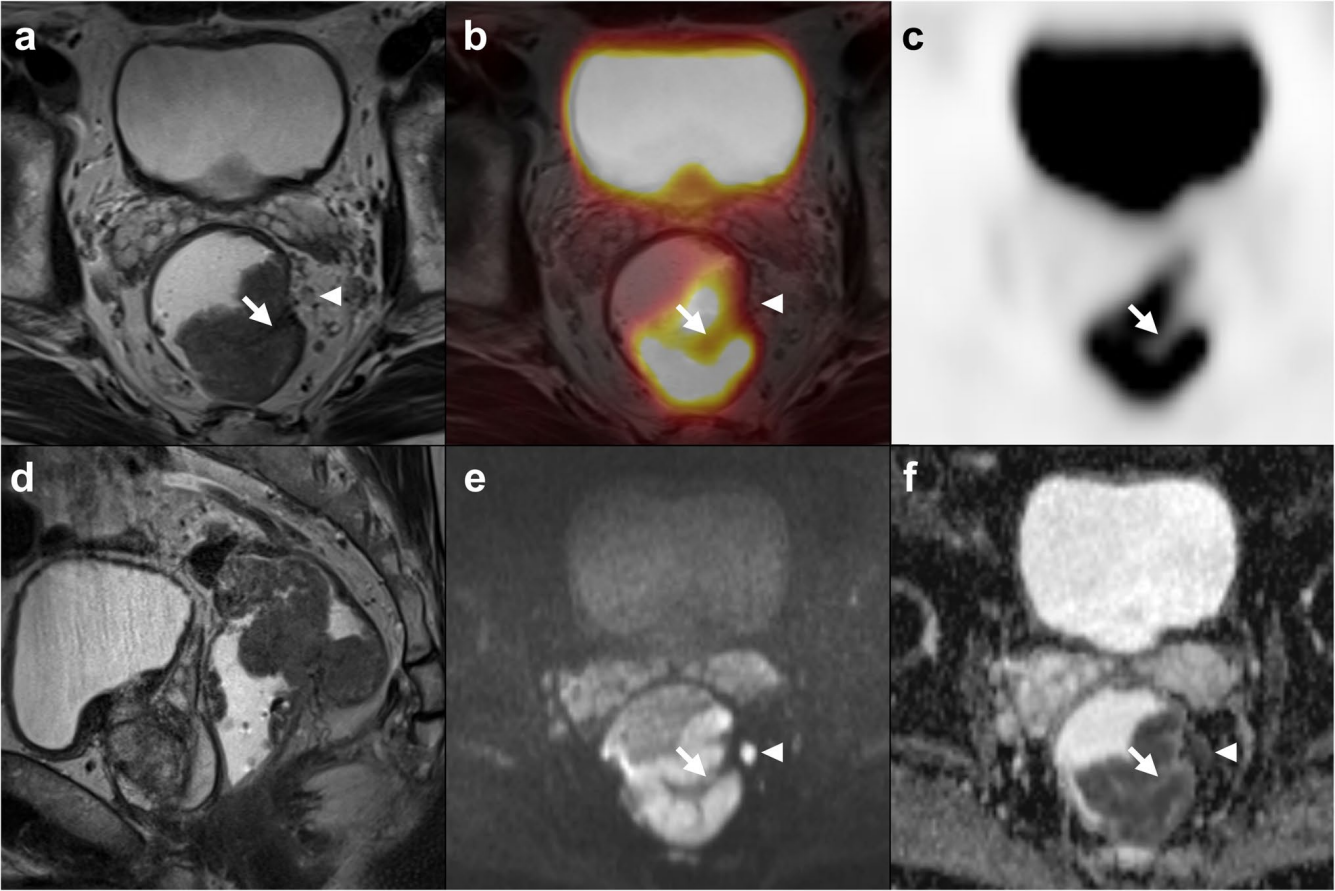
Axial (**a**) and sagittal (**d**) high-resolution T2w-MRI images, axial fused PET/MR (**b**), axial PET (**c**), axial diffusion-weighted imaging (DWI, b800) (**e**), and ADC map (**f**). Example of a pitfall and the necessity of careful examination of the anatomical and functional MRI images: Polypoid rectal cancer of the middle rectum with a high MTV (74.5 mL). T2w- and DWI images demonstrate a stalk without diffusion restriction or FDG-uptake (arrow), representing a primarily polypoid lesion. The rectal adventitia appears to be intact (mrT ≤ 2, despite high MTV). Arrowhead demonstrates a mesorectal lymph node, with equivocal MRI appearance and no FDG uptake. Histopathology confirmed pT2, N0

In previous studies, mainly relying on PET/CT, the PET component often failed to provide additional locoregional staging information. In our quantitative analysis of the PET data, MTV was independently associated with higher T-stage, as well as with LARC, similar to what is known from other tumor entities, such as cervical or esophageal cancer [33, 34]. Compared to MRI staging, MTV yielded an excellent sensitivity of 91.7% and specificity of 68.2% for LARC, significantly superior to anatomical MRI assessment. The MTV value might be used as an additional tumor feature to support the differentiation between borderline T2 and early T3 tumors, as demonstrated in our reported performance Figs. 3, 4, and 5. However, an independent confirmatory study in a different, prospective, patient cohort would be required before any clinical recommendation can be made. MTV also demonstrated an association with overall survival, indicating potential prognostic value, as has been proposed in previous studies [35]. This might contribute to the diagnostic value of volumetric PET parameters in the future. SUV_max_, SUV_peak_, and SUV_mean_ did not show any independent association with tumor stage, which reflects current data from PET/CT studies [36]. Furthermore, the early assessment of FDG uptake (30 vs. 60 min) did not show any benefit in gaining information on staging or prognosis compared to the standard acquisition.

The combined assessment of metabolic and functional imaging using PET/MRI is gaining attention in the evaluation of RC as a robust hybrid imaging technology because of its ability to combine high soft-tissue anatomic depictions with PET data in a single study. Queiroz et al. [37] evaluated PET and MRI parameters for the prediction of synchronous metastases in RC patients. In their study, the PET volumetric features TLG (352.9 vs 242.7, *P* = 0.046) and MTV (36.1 vs 26.2 mL, *P* = 0.03) significantly differed in patients with and without distant metastases and, thus, demonstrated higher values in patients with advanced tumors, while SUV_max_ and SUV_mean_ did not facilitate differentiation, strongly corresponding to our findings.

Other recent studies have mainly focused on comparison of PET/MRI to standard-of-care imaging and PET/CT, as well as the consequent impact on patient management. Only a few studies have investigated the diagnostic value of locoregional PET/MRI in the staging of untreated and treated patient cohorts. One study by Catalano et al. [25] found potential superior diagnostic accuracy of PET/MRI in the primary staging of untreated LARC, with PET/MRI outperforming MRI for evaluation of tumor length, external sphincter infiltration, and lymph node involvement, with a sensitivity of 92% and specificity of 86% vs. 88% and 43% for MRI, respectively. A composite reference standard of clinical, imaging follow-up, and histopathology was used as the gold standard for staging. Another study investigated the benefit of PET/MRI in the staging and restaging of RC, but did not demonstrate a significant improvement in PET/MRI versus MRI diagnostic accuracy for locoregional T- and N-staging compared to MRI [38]. However, in that study, a mix of treated and untreated patients rendered the reference standard of histopathology difficult to assess. In most diagnostic studies that include advanced RC, the reference standard is quite heterogenous, since it mostly consists of a combination of endoscopic biopsies, clinical and imaging follow-up, or surgical specimens after neoadjuvant CRT.

To the best of our knowledge, there has been no study investigating the diagnostic value of PET/MRI in the primary staging of untreated RC in a homogeneous patient cohort using the surgical specimen as a reference standard in each patient. In the authors’ opinion, this is the most reliable way to properly assess the true locoregional tumor stage and compare it to imaging performance.

### Limitations

Our study has several limitations. First, we performed a single-center retrospective study associated with a selection bias. A proportion of our patients had a low tumor stage with no unfavorable features. Advanced or metastatic cancers with higher rates of unfavorable features and survival events were excluded due to the essential need for neoadjuvant CRT, and thus, the inability to use histopathology as the gold standard. No patients with endangered or involved MRF were included in this study, hence limiting the relevance of these findings to earlier tumor stages.

Second, PET/MRI is still not yet widely implemented in the clinical routine in every imaging department. Although the routine use, where available, is quickly accepted by clinicians due to its comprehensive diagnostic information and its ability to function as a “one-stop shop” examination, the use of PET/MRI is still mainly restricted to referral centers.

Third, the longer acquisition times of PET/MRI entail lower patient throughput, and thus, higher costs. It might not be applicable in the fast-paced, economically driven setting of a non-academic imaging center.

Fourth, the analysis of PET and functional MRI parameters with dedicated software is complex and time-consuming, thus adding to the cost and time requirements of PET/MRI. The comparability of such results to other software solutions still must be clarified.

### Conclusion

PET/MRI as a multiparametric imaging tool improves the sensitivity of locoregional staging in LARC at a cost of specificity and, thus, could potentially influence preoperative treatment stratification.

## Supplementary Information

Below is the link to the electronic supplementary material.

Supplementary file1 (DOCX 19 KB)

Supplementary file2 (DOCX 18 KB)

## Abbreviations

PET/MRI: Positron emission tomography/magnetic resonance imaging
PET/CT: Positron emission tomography/computed tomography
mp: Multiparametric
FDG: 18F-Fluorodeoxyglucose
DWI: Diffusion-weighted imaging
RC: Rectal cancer
LARC: Locally advanced rectal cancer
MTV: Metabolic tumor volume
TLG: Total lesion glycolysis
TME: Total mesorectal excision
MRF: Mesorectal fascia
EMVI: Extramural vascular invasion
RT: Radiotherapy
CRT: Chemoradiotherapy
WB: Whole-body
VIBE: Volume-interpolated breath-hold
HASTE: Half-Fourier acquisition single-shot
TSE: Turbo spin echo
ADC: Apparent diffusion coefficient
LNR: Lymph node ratio
SUV_max_: Maximum standardized uptake value
SUV_peak_: Peak standardized uptake value
SUV_mean_: Mean standardized uptake value
OS: Overall survival
