# Supplementary material for: Added value of quantitative, multiparametric 18F-FDG PET/MRI in the locoregional staging of rectal cancer
Source: Eur J Nucl Med Mol Imaging. 2022 Sep 5;50(1):205–17. doi: 10.1007/s00259-022-05936-0 (PMC9668962; doi:10.1007/s00259-022-05936-0)
Supplement: Supplementary file 1 — Supplementary file1 (DOCX 19 KB) [file 259_2022_5936_MOESM1_ESM.docx]

**Supplemental material**

**Table S1 – Dedicated pelvic and WB PET/MRI protocol**

| **MR Sequence** | **Body part** | **Voxel Size (mm)** | **FOV (mm)** | **Matrix** | **Slice thickness (mm)** | **TR (msec)** | **TE (msec)** |
| --- | --- | --- | --- | --- | --- | --- | --- |
| Sagittal T2-w TSE | pelvis | 0.5x0.5x3.0 | 200 | 384x384 | 3 | 4000 | 104 |
| Coronal T2-w TSE | pelvis | 0.5x0.5x3.0 | 200 | 384x384 | 3 | 4000 | 104 |
| Axial T2-w TSE | pelvis | 0.6x0.6x0.5 | 200 | 320x320 | 5 | 3600 | 96 |
| Axial oblique T2-w TSE | pelvis | 0.5x0.5x3.0 | 200 | 384x384 | 3 | 4000 | 104 |
| DWI* | pelvis | 1.4x1.4x3.6 | 300 | 208x180 | 3.6 | 7000 | 92 |
| T1 VIBE Dixon fs | WB | 1.2x1.2x3.0 | 380 | 320x260 | 3 | 4.56 | 2.01 |
| T2 HASTE | WB | 1.5x1.5x6.0 | 380 | 256x256 | 6 | 1400 | 121 |
| DWI** | liver | 1.0x1.0x0.6 | 380 | 384x288 | 6 | 2000 | 73 |

| **PET** | **Image acquisition** | **Voxel size (mm)** | **Matrix** | **Slice thickness (mm)** | **Reconstruction algorithm** | **Iterations** | **Subsets** |
| --- | --- | --- | --- | --- | --- | --- | --- |
| pelvis | dynamic; 5 frames á 60 sec, 7 frames á 300 sec | 2.3x2.3x5.0 | 256x256 | 5 | HD-PET | 3 | 21 |
| WB | 4 positions á 180 sec | 2.3x2.3x5.0 | 172x172 | 5 | HD-PET | 3 | 21 |

WB, whole body; FOV, field of view; TR, repetition time; TE, time to echo, MR, Magnetic Resonance; HD-PET, High definition PET reconstruction; fs, fat saturation;

* b values 50, 800 s/mm^2^

** b values 50, 300, 600 s/mm^2^
