# Supplementary material for: Added value of quantitative, multiparametric 18F-FDG PET/MRI in the locoregional staging of rectal cancer
Source: Eur J Nucl Med Mol Imaging. 2022 Sep 5;50(1):205–17. doi: 10.1007/s00259-022-05936-0 (PMC9668962; doi:10.1007/s00259-022-05936-0)
Supplement: Supplementary file 2 — Supplementary file2 (DOCX 18 KB) [file 259_2022_5936_MOESM2_ESM.docx]

**Table S2 Diagnostic performance of MRI vs. multiparametric PET/MRI in the primary staging of RC compared to histopathology – other PET and MRI parameters**

| **PET/MRI parameters vs. histopathology** | | | | | |
| --- | --- | --- | --- | --- | --- |
| **Histopathological characteristics** | **PET/MRI parameters** | **Sensitivity** | **Specificity** | **PPV** | **NPV** |
| T-Stage (pT≥3a) | SUV_peak_ | 0.737 | 0.434 | 0.519 | 0.667 |
|  | SUV_mean_ | 0.895 | 0.348 | 0.531 | 0.80 |
|  | ADC | 0.75 | 0.308 | 0.545 | 0.538 |
| N-Stage (pN≥1) | SUV_peak_ | 0.545 | 0.567 | 0.269 | 0.733 |
|  | SUV_mean_ | 0.545 | 0.500 | 0.258 | 0.700 |
|  | ADC | 0.636 | 0.500 | 0.291 | 0.809 |
| LARC (pT≥3a, pN+) | SUV_peak_ | 0.739 | 0.474 | 0.629 | 0.60 |
|  | SUV_mean_ | 0.696 | 0.421 | 0.592 | 0.533 |
|  | ADC | 0.75 | 0.318 | 0.545 | 0.538 |

PPV, positive predictive value; NPV, negative predictive value; LARC, locally advanced rectal cancer
